# Editorial: Cardiorespiratory Coupling-Novel Insights for Integrative Biomedicine

**DOI:** 10.3389/fnins.2021.671900

**Published:** 2021-04-07

**Authors:** Maurizio Acampa, Andreas Voss, Tijana Bojić

**Affiliations:** ^1^Stroke Unit, Department of Emergency-Urgency and Transplants, Azienda Ospedaliera Universitaria Senese, “Santa Maria alle Scotte” General-Hospital, Siena, Italy; ^2^Institut für Innovative Gesundheitstechnologien (IGHT), Jena, Germany; ^3^Laboratory for Radiobiology and Molecular Genetics-080, Institute of Nuclear Sciences Vinča-National Institute of the Republic of Serbia, University of Belgrade, Belgrade, Serbia

**Keywords:** integrative physiology, cardiorespiratory coupling, autonomic nervous system, respiratory sinus arrhythmia, heart rate variability, arterial baroreflex, periodic repolarization dynamics, ProBNP

In recent years, integrative physiology is gaining increased attention; novel findings in the area of molecular and systemic cardiopulmonary interaction overgrow the classical opinion that the adoption and transport of oxygen and elimination of carbon dioxide are their only functions. Mechanical, molecular, endocrine, and neural subsystems of the autonomic nervous system are integrative scales of these two mutually dependent organs, providing a wider range of adaptation of the organism to the growing requirements of the environment, fitness, and pathological changes.

In the present Frontiers Research Topic, an international selection of investigators contributed original data to increase our current understanding about the complex cardiorespiratory interactions, providing novel findings about physiologic and pathogenic mechanisms and possible therapeutic advancement concerning the area of cardiorespiratory medicine.

Several contributions focused on new methods in order to properly investigate cardiorespiratory interactions, especially considering that cardiac and respiration signals have periodic oscillatory dynamics that can also be time-varying, so this increasing complexity have to take into account that frequency, coupling strength and coupling function are also varying in time.

In this view, Lukarski et al. developed a procedure for determination of the time window based on data analyses, as opposed to the previous practice of arbitrary choice. This study shows that the cardiorespiratory coupling strength and the similarity of form of coupling functions continuously change according to the breathing frequency, with greater values for slower breathing.

Cui et al. provided a further contribution in developing quantitative measures for respiratory sinus arrhythmia (RSA). RSA has profound significance in physiology and pathology and is usually evaluated by means of two techniques, the time and the frequency domain. By mathematically modeling this modulation, the authors proposed a quantitative measurement of RSA by means of the cardiopulmonary resonance function (CRF) and cardiopulmonary resonance indices (CRI) that are derived by disentanglement of the RR-intervals series into respiratory-modulation component (R-HRV), and non-respiratory component (NR-HRV), using Granger causality function. Their results suggest a superior representation ability of this technique in comparison with heart rate variability (HRV) and cardiopulmonary coupling index, with profound significance in physiology, pathology, and in possible future clinical applications.

The analysis of fluctuations in blood pressure and heart period represents another parameter of clinical importance as risk marker for cardiovascular morbidity and mortality (La Rovere et al., 2011), especially in some cardiac (Malberg et al., 2002) and non-cardiac diseases (Bär et al., 2007) or after specific therapeutic procedures (Acampa et al., 2011), that are able to determine changes at different levels, including arterial vascular walls, mechanosensitive ion channels, and voltage-gated ion channels (Tu et al., 2019). However, spontaneous baroreflex indices don't clearly reflect arterial baroreflex gain (Lipman et al., 2003); for this reason, Wessel et al. tested whether the xBRS method (Wesseling et al., 2017) is suitable to quantify the baroreflex sensitivity from non-invasive, non-interventional measurements under resting conditions. According to their analysis, xBRS method seems to have a potentially large bias in characterizing the capacity of the arterial baroreflex under resting conditions and seems to be exclusively dominated by the heart rate to systolic blood pressure ratio.

In this Frontiers topic, other studies focused on the important change of different cardiopulmonary parameters in different physiologic states such as wake and sleep, exercise and rest, circadian rhythms, as well as pathologic conditions.

One of these conditions, the deep sleep, is typically associated with an increased cardio-respiratory coupling, that corresponds to a maximal RSA. Based on that assumption, Zorko et al. evaluated HRV data, quantifying the (self)similarity among shapelets (that are short chunks of HRV time series), whose “shapes” are related to the respiration cycle; their results show distinctive patterns stable across age and sex, that are not only indicative of sleep and awake, but that are able to identify one more, potentially more sensitive indicator of sleep initiation. Additional studies are necessary that would involve contemporary classic polysomnography and novel, proposed index. In case of positive results, one of the possible applications of this approach could be related to public and general safety, by developing alarm systems that are able to recognize drowsiness in the person under the observation.

Another study by Limanskaya et al. examined a specific sleeping alteration, the central sleep apnea, by evaluating electroencephalogram, ECG, eye movements, air flow, thoracic respiratory muscle movements and myoelectric activity of the stomach and the duodenum in cats with sudden arrest of breathing during sleep. Their results suggest that the stereotypic coupling of activities in various visceral systems during episodes of central sleep apnea most likely reflects a complex adaptive behavior rather than an isolated respiratory pathology.

Another study by Matić et al. explored the physiological background of the non-linear operating mode of cardiorespiratory oscillators as the fundamental question of cardiorespiratory homeodynamics and as a prerequisite for the understanding of neurocardiovascular diseases. Their results show that cardiac and respiratory short-term and long-term complexity parameters have different state-dependent patterns supporting the hypothesis of a hierarchical organization of complexity regulatory mechanisms. In particular, a specific and comprehensive cardiorespiratory regulation in standing with 0.1 Hz breathing suggests that this state could represent the potentially most beneficial maneuver for cardiorespiratory conditioning, critically important for intensive care rehabilitation of artificially ventilated patients, as most actual in this moment- COVID 19 patients.

The interaction between breathing alterations and autonomic nervous system activity is another important subject, especially considering their possible pathogenic role in the pathogenesis of ventricular arrhythmias (Lai et al., 2019; Stavrakis et al., 2020); in this view, Schüttler et al. investigated the link among hyperventilation, sympathetic activity, and periodic repolarization dynamics (evaluated by means of beat-to-beat variations of the T wave vector on ECG). Their results suggest increased PRD values after hyperventilation, providing further insights about the alteration of ventricular repolarization associated to the hyperventilation.

Other two studies focused on the cardiopulmonary interactions during physical activity. Uryumtsev et al. investigated the mechanisms of oxygen supply regulation, which involves the respiratory and cardiovascular systems, during human adaptation to intense physical activity. Their results show that highly qualified athletes enhance intersystem integration in response to hypoxia, with a decreased oxygen consumption and a higher cardiorespiratory coherence in comparison with middle level athletes. In the second study, Abreu et al. evaluated healthy cyclist during and after inspiratory muscle training (IMT), that is a technique capable of improving cardiorespiratory interactions. In particular, they observed the effect of different degrees of IMT in amateur cyclists, analyzing electrocardiograms, non-invasive arterial pressures, and thoracic respiratory movements and quantifying cardiorespiratory coupling by means of squared coherence function, and causal model-based transfer entropy. In this way the authors demonstrated that the post-training increase of cardiorespiratory coupling might be the genuine effect of some rearrangements at the level of central respiratory network and its interactions with sympathetic drive and vagal activity.

The interaction between cardiorespiratory coupling and singing is the focus of another study of this Research Topic. Previous investigations about the effects of singing together focused on the synchronization of HRV, experienced by choir singers (Pearce et al., 2015). Ruiz-Blais et al. specifically evaluated HRV (using time-frequency coherence analysis) in pairs of non-experts in different vocalizing conditions; their results show that HRV becomes more coupled when people make long (>10 s) sounds synchronously and this synchronization persists when the effect of respiration is removed: these results suggest that since autonomic physiological entrainment is observed for non-expert singing, it may be exploited as part of interventions in music therapy or social prescription programs for the general population.

In this Research Topic other clinical studies show the relevance of the complex relationships between respiratory system and neurohormonal cardiac modulation involving both neural autonomic and humoral factors (hormonal factor, inflammatory cytokines). These complex mechanisms suggest particularly important implications in some diseases and during rehabilitation treatment. An unbalanced autonomic nervous system activity associated with different diseases can represent an important factor contributing to the occurrence of many specific cardiovascular complications (Goldberger et al., 2019). In this view, schizophrenia is a mental disorder that is associated with an increased cardiovascular mortality rate that could be determined by autonomic alterations (Laursen et al., 2014); on that basis, Schulz et al. assessed instantaneous cardiorespiratory couplings by quantifying the casual interaction between heart rate and respiration, in patients suffering from schizophrenia, compared with healthy first-degree relatives and control subjects. Their results clearly point to an underlying disease-inherent genetic component of the cardiac system for subjects with schizophrenia and first-degree relatives, while respiratory alterations seem to be only clearly present in patients with schizophrenia and correlated to their mental emotional states.

Obesity is another pathological condition that is associated with an increasing occurrence of cardiovascular complications even in childhood and adolescence: in this condition autonomic nervous system alterations can represent an important factor contributing to the initiation and progression of many cardiovascular disorders. However, the impaired parasympathetic control in obese patients seems to be associated with a different relative contribution of baroreflex and non-baroreflex (central) mechanisms underlying the origin of RSA. In particular, Javorka et al. applied a recently proposed information-theoretic methodology (partial information decomposition) to the time series of HRV, systolic blood pressure variability and respiration pattern, demonstrating that obesity is associated with blunted involvement of non-baroreflex RSA mechanisms and with a reduced response to postural stress (but not to mental stress).

The relationship between neural autonomic and humoral factors seems to be particularly important during rehabilitation of patients with chronic obstructive pulmonary disease (COPD): (Paulin et al., 2020) in a secondary analysis of a previous randomized trial (Paulin et al., 2017) point out for the first time for the relevance of Vitamin B12 on cardiovascular health in COPD subjects. Supplementation with vitamin B12 appears to lead to discrete positive effects on exercise tolerance in groups of subjects with more advanced COPD, significantly changing the time course of NT-proBNP responses during treatment. Even if their final analysis could not support a significant change in NT-proBNP levels owing to high-intensity constant work-rate exercise, the association between slower initial V'O2 adjustments toward a steady-state during rest-to-exercise transitions and more severe ventricular chamber volume/pressure stress recruitment, expressed by higher NT-proBNP secretion, suggest that vitamin B12 supplementation could modulate NT-proBNP secretion.

Furthermore, inflammatory markers can have a role in modulating autonomic nervous system in specific conditions, especially after surgery. It is well-known the association among cardiac and extracardiac surgery, inflammation and autonomic nervous system activity (Amar et al., 1998; Acampa et al., 2016). Clinical evidence shows that surgical procedures weaken the vagal tone, favoring a number of different complications such as sepsis, cardiac arrhythmias. In this view, Grote et al. demonstrated that orthopedic rehabilitation has the potential to strengthen the vagal activity and hence boost inflammatory control, also suggesting that a vagal reinforcement procedure prior to the surgery (“prehabilitation”) might be a beneficial strategy against post-operative complications.

In conclusion, the high-quality contributions of this Research Topic significantly enriched our knowledge about the field of Integrative Physiology, shedding light on complex physiologic and pathogenic mechanisms, with relevant clinical implications for patients' management. These studies also provide important suggestions for further investigation in this emerging area.

## Author Contributions

MA and TB contributed to the conception, design, and drafting of the work. TB and AV revised the draft. TB, AV, and MA approved the final version, agreed to be accountable for all aspects of the work, ensuring that questions related to the accuracy, or integrity of any part of it are appropriately investigated and resolved. All authors contributed to the article and approved the submitted version.

## Conflict of Interest

The authors declare that the research was conducted in the absence of any commercial or financial relationships that could be construed as a potential conflict of interest.

## References

[B1] Acampa M. Guideri F. Marotta G. Tassi R. D'Andrea P. Lo Giudice G.. (2011). Autonomic activity and baroreflex sensitivity in patients submitted to carotid stenting. Neurosci. Lett. 491, 221–226. 10.1016/j.neulet.2011.01.044 21262321

[B2] Acampa M. Lazzerini P. E. Guideri F. Tassi R. Martini G. (2016). Ischemic stroke after heart transplantation. J. Stroke 18, 157–168. 10.5853/jos.2015.01599 26915504 PMC4901943

[B3] Amar D. Fleisher M. Pantuck C. B. Shamoon H. Zhang H. Roistacher N.. (1998). Persistent alterations of the autonomic nervous system after noncardiac surgery. Anesthesiology 89, 30–42. 10.1097/00000542-199807000-00008 9667291

[B4] Bär K. J. Boettger M. K. Berger S. Baier V. Sauer H. Yeragani V. K.. (2007). Decreased baroreflex sensitivity in acute schizophrenia. J. Appl. Physiol. 102, 1051–1056. 10.1152/japplphysiol.00811.2006 17110512

[B5] Goldberger J. J. Arora R. Buckley U. Shivkumar K. (2019). Autonomic nervous system dysfunction: JACC focus seminar. J. Am. Coll. Cardiol. 73, 1189–1206. 10.1016/j.jacc.2018.12.064 30871703 PMC6958998

[B6] La Rovere M. T. Maestri R. Pinna G. D. (2011). Baroreflex sensitivity assessment – latest advances and strategies. Eur. Cardiol. Rev. 7:89. 10.15420/ecr.2011.7.2.89

[B7] Lai Y. Yu L. Jiang H. (2019). Autonomic neuromodulation for preventing and treating ventricular arrhythmias. Front. Physiol. 10:200. 10.3389/fphys.2019.00200 30914967 PMC6421499

[B8] Laursen T. M. Nordentoft M. Mortensen P. B. (2014). Excess early mortality in schizophrenia. Annu. Rev. Clin. Psychol. 10, 425–448. 10.1146/annurev-clinpsy-032813-153657 24313570

[B9] Lipman R. D. Salisbury J. K. Taylor J. A. (2003). Spontaneous indices are inconsistent with arterial baroreflex gain. Hypertension 42, 481–487. 10.1161/01.HYP.0000091370.83602.E6 12975383

[B10] Malberg H. Wessel N. Hasart A. Osterziel K. J. Voss A. (2002). Advanced analysis of spontaneous baroreflex sensitivity, blood pressure and heart rate variability in patients with dilated cardiomyopathy. Clin. Sci. 102, 465–473. 10.1042/cs1020465 11914109

[B11] Paulin F. V. Goelzer L. S. Müller P. T. (2020). Vitamin B12 supplementation and NT-proBNP levels in COPD patients: a secondary analysis of a randomized and controlled study in rehabilitation. Front. Neurosci. 14:740. 10.3389/fnins.2020.00740 32760247 PMC7372128

[B12] Paulin F. V. Zagatto A. M. Chiappa G. R. Müller P. T. (2017). Addition of vitamin B12 to exercise training improves cycle ergometer endurance in advanced COPD patients: a randomized and controlled study. Respir. Med. 122, 23–29. 10.1016/j.rmed.2016.11.015 27993287

[B13] Pearce E. Launay J. Dunbar R. I. (2015). The ice-breaker effect: singing mediates fast social bonding. Open Sci. 2:150221. 10.1098/rsos.150221 26587241 PMC4632513

[B14] Stavrakis S. Kulkarni K. Singh J. P. Katritsis D. G. Armoundas A. A. (2020). Autonomic modulation of cardiac arrhythmias: methods to assess treatment and outcomes. JACC Clin. Electrophysiol. 6, 467–483. 10.1016/j.jacep.2020.02.014 32439031 PMC7370838

[B15] Tu H. Zhang D. Li Y. L. (2019). Cellular and molecular mechanisms underlying arterial baroreceptor remodeling in cardiovascular diseases and diabetes. Neurosci Bull. 35, 98–112. 10.1007/s12264-018-0274-y 30146675 PMC6357267

[B16] Wesseling K. H. Karemaker J. M. Castiglioni P. Toader E. Cividjian A. Settels J. J.. (2017). Validity and variability of xBRS: instantaneous cardiac baroreflex sensitivity. Physiol. Rep. 5:e13509. 10.14814/phy2.13509 29180481 PMC5704083

